# SFV Replicon Vector Harbouring Porcine Epidemic Diarrhoea Virus Immunogens Delivered by Attenuated *Salmonella* Typhimurium Induces PEDV Neutralising Antibodies and Lactogenic Immunogenicity in BALB/c Mice

**DOI:** 10.3390/v18030375

**Published:** 2026-03-17

**Authors:** Chamith Hewawaduge, Ji-Young Park, Jaime C. Cabarles, Gayeon Won, John Hwa Lee

**Affiliations:** 1 Sri Lanka Institute of Biotechnology, Pitipana, Homagama 10200, Sri Lanka; 2 Division of Zoonotic and Vector Borne Disease Research, National Institute of Health, Korea Disease Control and Prevention Agency, Osong, Cheongju 28160, Republic of Korea; 3 College of Agriculture, Resources and Environmental Science, Central Philippine University, Jaro, Iloilo City 5000, Philippines; jccabarlesjr@cpu.edu.ph; 4 College of Veterinary Medicine, Jeonbuk National University, Iksan Campus, Iksan 54596, Republic of Korea

**Keywords:** *Salmonella* Typhimurium, PED virus, salmonellosis, humoral and cell-mediated immunity, neutralising antibodies, lactogenic immunity

## Abstract

Background: Porcine epidemic diarrhoea virus (PEDV) is a highly contagious pathogen causing severe diarrhoea and high mortality in neonatal piglets. Methods: In this study, consensus sequences encoding the N-terminal domain of spike subunit 1 (S1-NTD) and nucleocapsid (N) protein of PEDV were cloned into a eukaryotic expression vector pJHL204 and transformed into an attenuated *Salmonella* Typhimurium strain JOL2500. Antigen expression was confirmed by Western blot and immunofluorescence analyses. The recombinant strains were evaluated in vivo for safety, persistence, and immunogenicity. Immunogenicity was characterised by measuring antibody response, virus neutralising assays, cytokine profiling, and flow cytometric analysis of T cell subpopulation. Protective efficacy against salmonellosis in dams and passive transfer of neutralising antibodies to suckling mice were evaluated. Results: Vaccinated mice exhibited no adverse effects or bacterial persistence in major organs, confirming the vaccine’s safety. Immunisation elicited robust PEDV- and *Salmonella*-specific humoral and cell-mediated immune responses. Upon *Salmonella* challenge, vaccinated mice showed significantly reduced bacterial loads in splenic tissues. Furthermore, vaccinated dams and their offspring induced detectable anti-PEDV neutralising antibodies, indicating successful passive antibody transfer. Conclusion: Our findings indicate that the designed vaccine constructs provide a promising platform for inducing multifaceted immuno-protectivity against PEDV and salmonellosis.

## 1. Introduction

Porcine epidemic diarrhoea (PED) is a highly infectious epizootic disease that occurs in swine of any age and poses a substantial economic burden to the poultry industry worldwide [1,2,3]. PED infection is associated with higher morbidity in fattening and suckling piglets, whereas suckling piglets are the most vulnerable to the disease, with a 50–90% mortality rate. Therefore, vaccine-induced lactogenic immunity is vital in acquiring passive protection of piglets against PED. Even though commercial vaccines have demonstrated efficacy in experimental conditions [4], the protective immunity elicited by these vaccines appears to be inadequate under field conditions [5,6]. The PEDV S protein is an enveloped glycoprotein that plays a pivotal role in virus attachment [7]. Notably, the N-terminal domain of the PEDV S1 subunit serves as the receptor-binding domain and induces a high neutralising antibody titer [8,9], making it a key target for vaccine design. Additionally, the nucleocapsid (N) protein is a highly conserved, multi-functional structural protein involved in viral replication and pathogenesis, harbouring several antigenic epitopes essential for immune recognition [10]. Furthermore, clinical cases of salmonellosis caused by antimicrobial-resistant *Salmonella* Typhimurium (*S*. Typhimurium) DT104 have been increasingly reported in pig farms, posing a significant threat to both animal health and human food safety [11]. This highlights an urgent need for developing vaccines that can confer broad protection against circulating PEDV strains while addressing the threat of salmonellosis in the swine population.

Current PEDV vaccines face significant limitations, including reversion to virulence, poor cross-protection, and inadequate mucosal/lactogenic immunity. A critical challenge in PEDV control is the protection of neonatal piglets, whose survival depends almost exclusively on the passive transfer of maternal antibodies through colostrum and milk [12,13]. While many vaccine strategies struggle to achieve sufficient antibody titers in mammary secretions, the use of live bacterial vaccine vectors (LBVs) via intramuscular administration provides a robust alternative to traditional inactivated or subunit vaccines [14]. LBVs such as attenuated *Salmonella* have been employed to deliver both homologous and heterologous antigens to stimulate mucosal and systemic immune responses [15,16].

Cultivating the virulent genes through genetic means can attenuate live *Salmonella* vectors, making them non-pathogenic while preserving their immunogenicity. Deletion of the *lon* and *cpxR* genes enhances bacterial invasiveness by modulating crucial early host cell entry and promoting systemic dissemination [17]. This genetic alteration prompts the host cell’s oxidative defence mechanisms, facilitating intracellular clearance of the vector. Additionally, the *sifA* gene on the *Salmonella* pathogenicity island 2 (SPI-2) plays a crucial role in preserving the membrane integrity of *Salmonella*-containing vacuoles (SCVs) [18]. Absence of the *sifA* gene leads to bacterial escape into the host cytosol, resulting in augmented major histocompatibility complex (MHC) class I antigen presentation and initiating NK cell activation [19,20]. Unlike conventional DNA-based bacterial delivery systems that often suffer from in vivo protein expression, the self-amplifying RNA mechanism ensures high-level cytoplasmic mRNA replication [21]. To achieve efficient transcription and translation of antigen in eukaryotic cells, we employed an expression plasmid, PJHL204, which encodes the Semliki Forest virus (SFV) RNA-dependent RNA polymerase (RdRp) [22]. This system facilitates mRNA amplification in the host cell cytoplasm, thereby significantly improving antigen production and immunity. By combining the systemic robustness of IM delivery with the high expression efficiency of the SFV replicon, this platform acts as a potent endogenous adjuvant that maximises the induction of high-titer lactogenic immune responses, addressing the limitations of low immunogenicity and inadequate field protection observed in current PEDV vaccine candidates.

The primary objective of this study was to develop and evaluate a novel vaccine platform utilising an attenuated ∆*lon*, ∆*cpxR*, and ∆*sifA S. * Typhimurium strain as a systemic delivery vector for an SFV replicon-based DNA vaccine encoding PEDV S1 and N antigens. Specifically, we aimed to (1) construct and characterise an attenuated *Salmonella* Typhimurium strain delivering pJHL204-SFV replicon plasmids encoding the PEDV S1(19-223a.a) and N protein for eukaryotic expression and antigen presentation; (2) conduct a safety assessment of the vaccine constructs by monitoring physiological changes, performing histopathological examinations, and enumerating colony forming units in major organs; (3) evaluate intramuscular vaccination induced humoral and cell-mediated immune responses against both PEDV and *Salmonella* antigens in dams; (4) assess lactogenic immunity to suckling pups via antibody quantification and neutralisation assays; and (5) demonstrate heterologous protection against wild-type *Salmonella* challenge in a mouse model, thereby addressing two major swine pathogens within a single intervention.

## 2. Materials and Methods

### 2.1. Bacterial Strains, Plasmids, Cell Lines, and Viruses

The bacterial strains and plasmids used in this study are listed in Table 1 and were selected based on their well-characterised genetic backgrounds and suitability for their experimental applications. *S*. Typhimurium JOL401, a wild-type strain possessing intact *Salmonella* pathogenic islands (SPI-1 through SPI-5), was utilised to ensure immunological relevance reflecting the natural virulence factor repertoire. The *E. coli* X232 strain was utilised for subcloning synthetic PEDV genes into the balanced lethal *asd*+ plasmid pJHL204 due to *recA1* and *endA1* mutations, which minimise recombination and plasmid degradation, alongside the ∆*asd* mutation that complements the *asd*- auxotrophy of the *Salmonella* delivery vector JOL2500 for plasmid stability without antibiotic selection. Similarly, BL21(pLysS) was used in this study, capable of inducing overexpression of His-tagged PEDV antigens from PET28a, owing to its protease-deficient *ompT* and *lon* backgrounds, T7 RNA polymerase system for tight regulation, and pLysS lysyl-tRNA supplement to suppress basal toxicity, enabling efficient purification of polyclonal antibodies for downstream immunogenicity assays. Attenuated *Salmonella* and *Escherichia coli* strains were grown at 37 °C in Luria–Bertani (LB) broth (BD BIosciences, Franklin Lakes, NJ, USA) with or without appropriate antibiotics unless otherwise indicated. A total of 50 µg/mL of diaminopimelic acid (DAP) (Sigma-Aldrich, St. Louis, MO, USA) was added for the growth of Δ*asd* strains [23]. The tissue culture infective dose (TCID50) of the PED virus was propagated in African green monkey kidney (Vero) cells (ATCC CCL-81, Manassas, VA, USA), as described previously [24].

### 2.2. Construction and Validation of Attenuated Salmonella Delivering Immunogenic PEDV Antigens

The synthetic genes encoding PEDV S1-NTD (19-223a.a) and N protein, codon optimised for eukaryotic expression, were commercially synthesised by Cosmogenetech (Seoul, Republic of Korea) based on consensus sequences derived from multiple alignments of 47 South Korean PEDV isolates (2010–2022) using ClustalW (EMBL-EBI). These sequences were selected for their high conservation (>95% identity), immunogenicity (predicted by identifying T and B cell epitopes (IEDB epitope mapping)), and inclusion of neutralising domains. The genes were directionally subcloned into an *asd*+ balanced lethal vector, pJHL204, via restriction digestion and T4 ligation, verified by sequencing. Plasmids were then electroporated (2.5 kV, 25µF, 200Ω) into electrocompetent attenuated *S*. Typhimurium strain JOL2500. Transformants were selected on LB-DAP (50 µg/mL) agar, confirmed by colony PCR and plasmid miniprep/restriction profiling, yielding strains JOL2669 (pJHL204-S1-NTD (19-223a.a)) and JOL2670 (pJHL204-N). Plasmid stability was assessed by 50 serial passages in non-selective LB-DAP broth, retaining >95% transformants as verified by PCR. For protein production, antigens were subcloned into the pET28a (+) expression vector and transformed into *E. coli* BL21 (DE3) pLysS, induced with 1mM IPTG, and purified via Ni-NTA chromatography (Qiagen, Hilden, Germany). Expression was induced with 1mM IPTG, followed by Ni-NTA affinity purification. Western blotting with anti-His antibodies and SDS-PAGE analysis confirmed the expression of the purified proteins. Polyclonal antibodies against each antigen were raised in specific pathogen-free New Zealand white rabbits.

### 2.3. Western Blot and Immunofluorescence Assay

The expression of each antigen was confirmed by Western blot and immunofluorescence analysis. RAW 264.7 cells were grown in 12-well plates and grown to 80% confluency in DMEM (Lonza, Basel, Switzerland) supplemented with 10% FBS (Gibco, Waltham, MA, USA) at 37 °C and 5% CO_2_. Cells were infected with strains JOL2669, JOL2670, and JOL2865 (vector control) at an MOI of 50 for 4 h, followed by gentamicin treatment (100 µg/mL) for 1 h to eliminate extracellular bacteria, and incubated for 48 h. For Western blot analysis, cells were prepared in RIPA buffer supplemented with protease inhibitors, kept on ice for 15 min, and sonicated. Lysates (30 µg) were resolved by SDS-PAGE on 12% gels, transferred to PVDF membranes, and blocked with 5% skim milk in 1× TBST. Membranes were probed overnight at 4 °C with rabbit polyclonal anti-S1-NTD or anti-N primary antibodies (1:1000 dilution), followed by HRP-conjugated anti-rabbit IgG (Southern Biotech, Birmingham, AL, USA) at 1:6000 dilution for 1 h at room temperature. Expression of each antigen was detected by chemiluminescence (ECL reagent, Cytiva, Marlborough, MA, USA) using a ChemiDoc imaging system (Cytiva, Marlborough, MA, USA).

For the immunofluorescence assay, following 48 h post-infection, cells were fixed with 4% paraformaldehyde for 15 min at room temperature and blocked with 5% BSA for 1 h at room temperature. Cells were then incubated with primary antibodies, anti-S1-NTD or anti-N, at a dilution of 1:1000 at 4 °C overnight, followed by incubation with Alexa Fluor-488 anti-rabbit IgG (1:1000) (Invitrogen, Thermo Fisher Scientific, Waltham, MA, USA) secondary antibody for 1 h at room temperature. Cells were washed three times with PBS, examined, and imaged using the Leica Fluorescence Microscope (Leica Biosystems, Wetzlar, Germany).

### 2.4. Safety Assessment of the PED Vaccine Constructs in Mice

Five-week-old female BALB/c mice (Samtako, Osan, Republic of Korea) were acclimatised for 7 days and individually identified by ear tags. Animals were stratified by body weight (20.1 ± 1.2 g) and allocated to experimental groups using stratified block randomisation implemented in GraphPad Prism 9. Group allocation sequences were generated by an independent, blinded technician. All personnel performing intramuscular vaccinations, bleedings, and immunological and histopathological assays remained blind to group identities. All animal experimental procedures were approved (IACUC, NON2022-024-003) by the Jeonbuk National University Animal Ethics Committee following the guidelines of the Korean Council on Animal Care and Korean Animal Protection Law, 2007; Article 13 (Experiments with animals). Mice were housed in ventilated cages provided with sterile water and food ad libitum, with 12 h light-dark cycles.

To assess the safety of the PED vaccine constructs, six-week-old female BALB/c mice (*N* = 125) were randomly divided into three groups and administered via the intramuscular route (Table 2). Throughout the experiment, the mice were closely monitored for changes in body weight, clinical signs, and mortality. On days 1, 3, 5, 7, 14, and 21 post-inoculation, five mice from each group were humanely euthanised, and liver and spleen samples were collected. A portion of each sample was preserved in 10% neutral-buffered formalin and processed for histopathological examination. The remaining samples were weighed, homogenised in 1 mL of buffered peptone water, and plated onto brilliant green agar (BGA) plates to enumerate colony-forming units (CFUs).

### 2.5. Immunisation and Challenge

Seventy-five six-week-old female BALB/c mice were randomly divided into five groups (n = 15) and immunised with a prime booster strategy according to the scheme presented in Table 3. Serum samples were collected from the retro-orbital sinus of each mouse on the 14th day post-booster immunisation. To evaluate the T cell immune response elicited by the vaccine constructs, five mice from each group were euthanised on the 14th day post-booster immunisation, and splenocytes were aseptically collected for flow cytometry (FACS) and quantitative real-time PCR (qPCR) assays.

To determine the protective efficacy against salmonellosis induced by vaccination, in the sixth week post-primary vaccination, five mice from each group were challenged intraperitoneally with the wild-type *Salmonella* strain (JOL401) at a dose of 1 × 10^5^ cells/100 µL. The challenged mice were monitored daily for clinical signs, mortality, and weight loss. On the fifth day post-challenge, spleens were collected, weighed, and homogenised, and the bacterial load was enumerated in log CFU/mL. Spleens sampled from challenged mice were fixed in 10% formalin and subjected to H&E staining for histopathological analysis.

### 2.6. Determination of Antigen-Specific ELISA

To assess the anti-PEDV and anti-*Salmonella* antibody response induced by vaccination, humoral immune responses were evaluated by indirect ELISA. Briefly, 96-well ELISA plates were coated with purified S1-NTD, N, and *Salmonella* outer membrane proteins (300 ng/well) in carbonate–bicarbonate coating buffer (9.6 pH) and incubated overnight at 4 °C. Serum samples were serially diluted, added to test wells, and incubated for 1 h at 37 °C. After washing the plates with PBST (0.5% Tween-20), IgG, IgG1, and IgG2a titers against PEDV and Salmonella antigens were quantified by incubating with HRP-conjugated goat anti-mouse IgG, IgG1, or IgG2a secondary antibodies (Southern Biotech, Birmingham, AL, USA) at a 1:3000 dilution for 1 h at 37 °C. After final washing with PBST, OPD substrate (Sigma-Aldrich, St. Louis, MO, USA) was used to develop, and the OD values were read at 492 nm using an ELISA plate reader (Tecan, Männedorf, Switzerland).

### 2.7. Cytokine Responses

Spleens were aseptically harvested from immunised mice on day 14 booster, mechanically dissociated in RPMI 1640 (Gibco, USA) using 70 µm cell strainers (SPL life sciences, Pocheon, Republic of Korea), and lysed with 1X ACK buffer (Lonza, Switzerland) for 5 min at room temperature. Splenocytes were washed and resuspended in complete RPMI 1640 medium supplemented with 10% FBS and 1% penicillin/streptomycin (Gibco, USA). Cells were seeded in 6-well plates (SPL Life Sciences, Republic of Korea) at a density of 5 × 10^6^ cells/well and stimulated with recombinant PEDV S1-NTD (2 µg/well) and PEDV-N (2 µg/well) proteins for 48 h. An unstimulated control was included in the assay. Cells were lysed in RiboEx buffer (GeneAll Biotechnology, Seoul, Republic of Korea), and RNA was extracted according to the manufacturer’s protocol (GeneAll^®^ Hybrid-R™ kit, GeneAll Biotechnology, Republic of Korea). RNA was quantified by the Infinite M Nano absorbance microplate reader (Tecan, Switzerland). Complementary DNA (cDNA) was prepared from 1 µg of RNA using ReverTra Ace^®^ qPCR RT Master Mix (TOYOBO, Osaka, Japan), and samples were stored at −20 °C until analysis. Levels of IFN-γ and IL-4 mRNA were analysed by qPCR using the primers listed in Table 1. Real-time PCR was carried out using EzAmp™ RT-qPCR 2X Master Mix (Elpis Biotech, Daejeon, Republic of Korea) according to the manufacturer’s instructions and performed in the Applied Biosystems^®^ StepOnePlus Real-Time PCR system. Relative gene expression levels of IL-4 and INF-γ mRNA were normalised to β-actin and determined by the 2^−∆∆CT^ method [26].

### 2.8. FACS Analysis of CD3^+^CD4^+^ and CD3^+^CD8^+^ T Cell Populations

Splenocytes were isolated as described above (Section 2.7) from immunised mice on the 14th day after booster vaccination. For stimulation, 2 × 10^5^ viable cells/well were seeded in round-bottom 96-well plates (SPL life sciences, Republic of Korea) in complete RPMI 1640 medium and stimulated with recombinant PEDV S1-NTD (1 µg/well) and PEDV-N (1 µg/well) proteins for 72 h at 37 °C, 5% CO_2_. Fc Block solution was prepared using anti-CD16 (Miltenyi Biotec, Bergisch Gladbach, Germany) in FACS buffer, and cells will be incubated for 15 min at 4 °C. The cells were then stained with a cocktail containing anti-mouse CD3a-PE (Miltenyi Biotec, Germany), CD8-FITC (Miltenyi Biotec, Germany), and CD4-perCP-vio700 (Miltenyi Biotec, Germany) and incubated for 30 min at 4 °C in the dark. The proportions of CD4^+^ and CD8^+^ T cell subpopulations were determined using a MACSQuant^®^ analyser (Miltenyi Biotec, Germany). Data acquisition and analysis were performed using MACSQuantify™ (Miltenyi Biotec, Germany) software, acquiring 1 × 10^5^ events/sample. Lymphocytes were identified based on the forward scatter (FSC) and side scatter (SSC) dot plot of the whole splenocyte cell suspension. Live cells were gated as DAPI negative. T cell activation was quantified as CD4^+^/CD8^+^ percentages and mean fluorescence intensity (MFI) relative to unstimulated controls within each group.

### 2.9. Serum Neutralisation Test

Sera collected from mice on day 14 post-booster immunisation were heat-inactivated at 56 °C for 30 min. The sera were serially diluted in two-fold increments, mixed with an equal volume of PEDV suspension containing 200 TCID50, and incubated at 37 °C for 1 h. Following incubation, serum-virus mixtures were inoculated onto a Vero cell monolayer and incubated for 1 h at 37 °C in a 5% CO_2_ incubator, and cells were overlayed with 2% FBS containing DMEM and incubated further in the 5% CO_2_ incubator. Cytopathic effect (CPE) was observed daily, and neutralising antibody titers were calculated using the Reed–Muench method.

### 2.10. Evaluation of PEDV-Specific Lactogenic Immunity in Suckling Mice

Following booster immunisation, adult female mice were mated with male mice, and passive immunity was evaluated in suckling mice by PEDV antigen-specific ELISA. During the second week of lactation, pups were sacrificed, and blood and intestinal samples were collected (N = 20, n = 5). Pup intestinal IgA was assessed by collecting the lavage of the small intestine. Briefly, collected intestinal samples were flushed with 500 μL PBS + 0.1% Tween-20 + protease inhibitors, homogenised, and centrifuged. The serum and intestinal supernatant samples were then used to validate antigen-specific IgG and IgA ELISA, respectively. Additionally, PEDV serum neutralisation titer was determined according to the protocol described above.

### 2.11. Statistical Analysis

All data were statistically analysed using GraphPad Prism 9 software and expressed as mean ± standard deviation. For survival curves, the Kaplan–Meier survival analysis method was implemented.

## 3. Results

### 3.1. Evaluation and Characterisation of the PEDV Antigens

**Figure 1:**
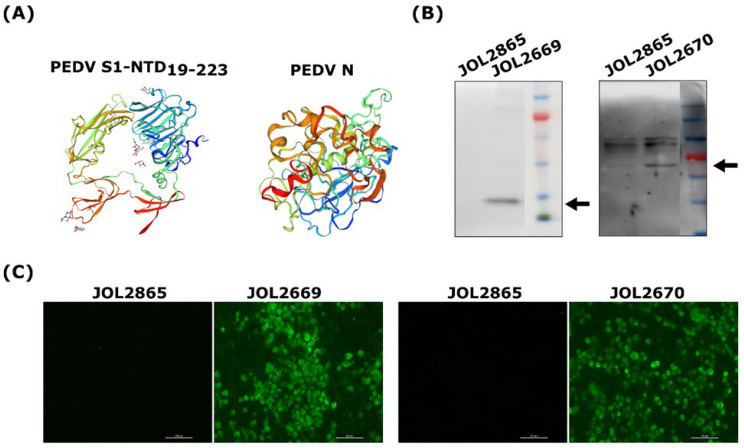
Characterisation of recombinant PEDV antigens. (**A**) In silico prediction of the tertiary structures of modelled PEDV S1-NTD19-223 and PEDV N antigens. Following bactofection of RAW 264.7 cells with JOL2669 and 2670 strains, expression of antigens was validated by (**B**) Western blot analysis, where the arrows represent the expected reactive bands at 28 kDa and 58 kDa in the recombinant strains JOL2669 and JOL2670, and (**C**) Immunofluorescence assay (IFA) using polyclonal antibodies raised against each target protein. Scale bar = 50 μm.

The three-dimensional (3D) structures of the vaccine antigens, comprising the S1-NTD and N proteins of PEDV, were constructed using SWISS-MODEL (https://swissmodel.expasy.org/; accessed on 6 May 2022). Structural comparison revealed that the S1-NTD and N protein models share 89% and 91% homology, respectively, with the corresponding proteins of the PEDV parental strain, respectively (Figure 1A). Antigenic profiling using the IEDB Analysis Resource (http://tools.iedb.org/; accessed on 6 May 2022) predicted key immune epitopes within these proteins. For S1-NTD, amino acids from 46 to 60 and 156–170 are predicted to be involved in CD4^+^ T cell immune responses, and amino acids from 39 to 47 are predicted to be involved in MHC-I binding. Similarly, for the N protein, amino acids from 56 to 77 and 121–135 are involved in CD4^+^ T cell immune responses, and amino acids from 156 to 165 are predicted to be associated with MHC-I binding (Figure 1A). These findings suggest that the selected vaccine antigens harbour epitopes capable of eliciting both helper and cytotoxic T cell responses, proving their potential efficacy in inducing cellular immune responses against PEDV.

### 3.2. Construction and Validation of the PEDV Antigens Expressed by Attenuated S. Typhimurium

The conserved sequences of S1-NTD and N antigens of circulating PEDV strains were selected, synthesized, and cloned into an SFV replicon vector, pJHL204. The expression of the vaccine antigens was confirmed by Western blot and immunofluorescence assays. As shown in Figure 1, expected reactive bands were detected in the cell lysate of recombinant strains JOL2669 and JOL2670 at 28 kDa and 58 kDa, respectively (Figure 1B). Furthermore, an intense emission of green fluorescence was observed in cells infected with PED vaccine strains JOL2669 and JO2670 (Figure 1C). These results confirm that antigen expression was driven by RNA-dependent RNA polymerase activity of the SFV replicon, amplifying the mRNA encoding the PEDV proteins of interest and enabling robust protein production.

### 3.3. Evaluation of the Safety of the Attenuated S. Typhimurium Strain Delivering PED Vaccine Antigens

**Figure 2:**
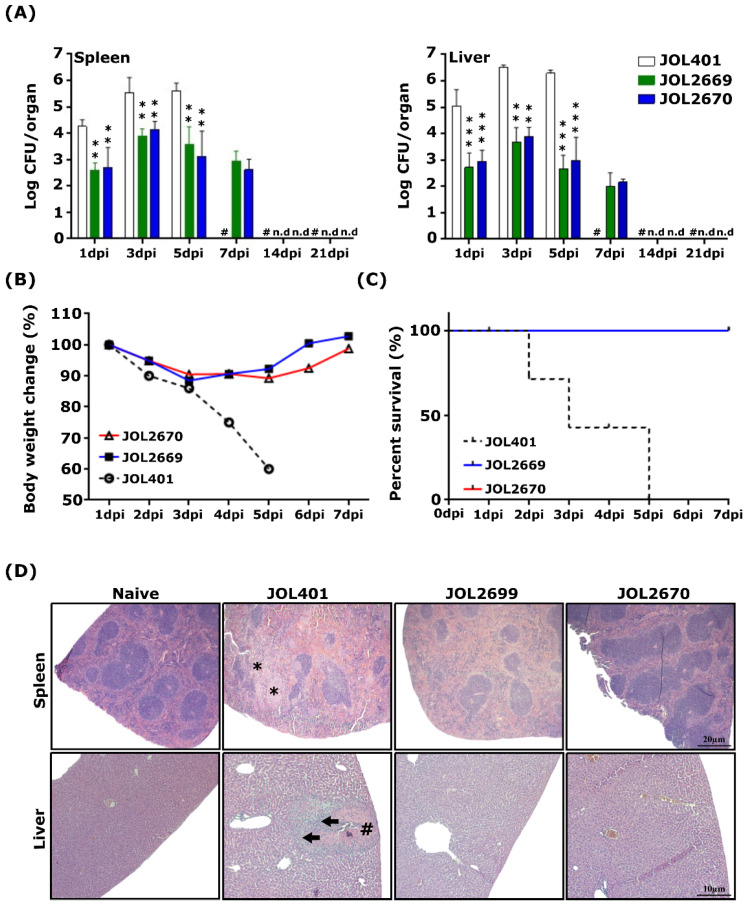
Post-immunisation safety parameters. Mice were immunised with JOL401, JOL2669, and JOL2670 via the intramuscular route. (**A**) Following immunisation, spleen and liver samples were aseptically collected and weighed. Mice immunised with JOL2669 and JOL2670 showed no bacterial recovery from organs collected on the 14th and 21st day post-inoculation (dpi). Data are presented as mean ± SD and were analysed by multiple unpaired *t*-tests. (**B**) Bodyweight change and (**C**) survival rate were noted up to 7 days post-inoculation. Mice inoculated with JOL401 demonstrated dramatic body weight reduction and 100% mortality by 5 dpi. (**D**) Histopathology (H&E). The spleen from JOL401-inoculated mice exhibited white pulp atrophy (*), while the liver showed coagulative necrosis (#) and mononuclear cell infiltration (arrows). ** *p* ≤ 0.01, *** *p* ≤ 0.001. n.d: not detected.

The safety of the attenuated *Salmonella* strain delivering antigens against PEDV was assessed and compared with its wild-type strain. The ST mutant strain was found to successfully colonise the spleen and liver of immunised mice up to day 7 post-inoculation and was completely cleared by day 14 (Figure 2A) (Supplementary Table S1). A dramatic reduction in body weight was observed in mice inoculated with the wild-type strain, and 100% mortality was observed by the 5th day post-inoculation. Importantly, no mortality or notable body weight reduction was observed in mice inoculated with recombinant *Salmonella* strain (Figure 2B,C). Histopathological observations revealed that white pulp atrophy in splenic tissues and mononuclear cell infiltration in the liver were observed in mice inoculated with the JOL401 strain. Conversely, no significant tissue alterations were observed in mice inoculated with the attenuated *Salmonella* strain (Figure 2D). These findings confirm the markedly reduced virulence and enhanced safety of the attenuated *Salmonella* strain while maintaining transient organ colonisation necessary for effective antigen delivery.

### 3.4. Vaccination-Induced Anti-PEDV Specific Humoral Immune Responses

**Figure 3:**
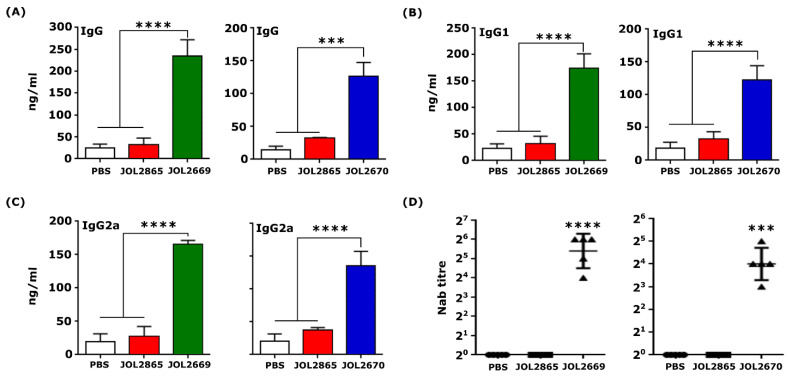
Humoral immune response in mice immunised with JOL2669 and JOL2670 strains during the second week post-booster immunisation. PEDV antigen-specific (**A**) IgG, (**B**) IgG1, and (**C**) IgG2a were measured by ELISA. (**D**) Detection of neutralising antibody titers in the serum of immunised mice was performed using the serum neutralisation (SN) assay. *** *p* ≤ 0.001, **** *p* ≤ 0.0001.

Serum samples from immunised and control mice were subjected to indirect ELISA. Compared to the vector control group, mice immunised with JOL2669 exhibited a 7.08-fold increase in IgG levels (*p* < 0.0001), while those immunised with JOL2670 showed a 3.9-fold increase (*p* < 0.001) (Figure 3A). Further analysis of IgG subclasses revealed elevated IgG1 and IgG2a in both vaccine groups, indicating induction of balanced Th1/Th2 immune responses (Figure 3B,C). These data demonstrate that the *Salmonella*-based PED vaccine constructs effectively activate humoral immunity with mixed Th1/Th2 polarisation, a critical immune profile for PEDV vaccines.

Subsequently, serum neutralising assays confirmed that mice vaccinated with JOL2669 and JOL2670 elicited significantly higher neutralising antibody titers of 6 log2 and 4 log2, respectively (Figure 3D). These neutralisation profiles validated that the selected PEDV antigens contain neutralising epitopes, emphasising the protective potential of the vaccine candidates.

### 3.5. Determination of Cytokine Measurement

**Figure 4:**
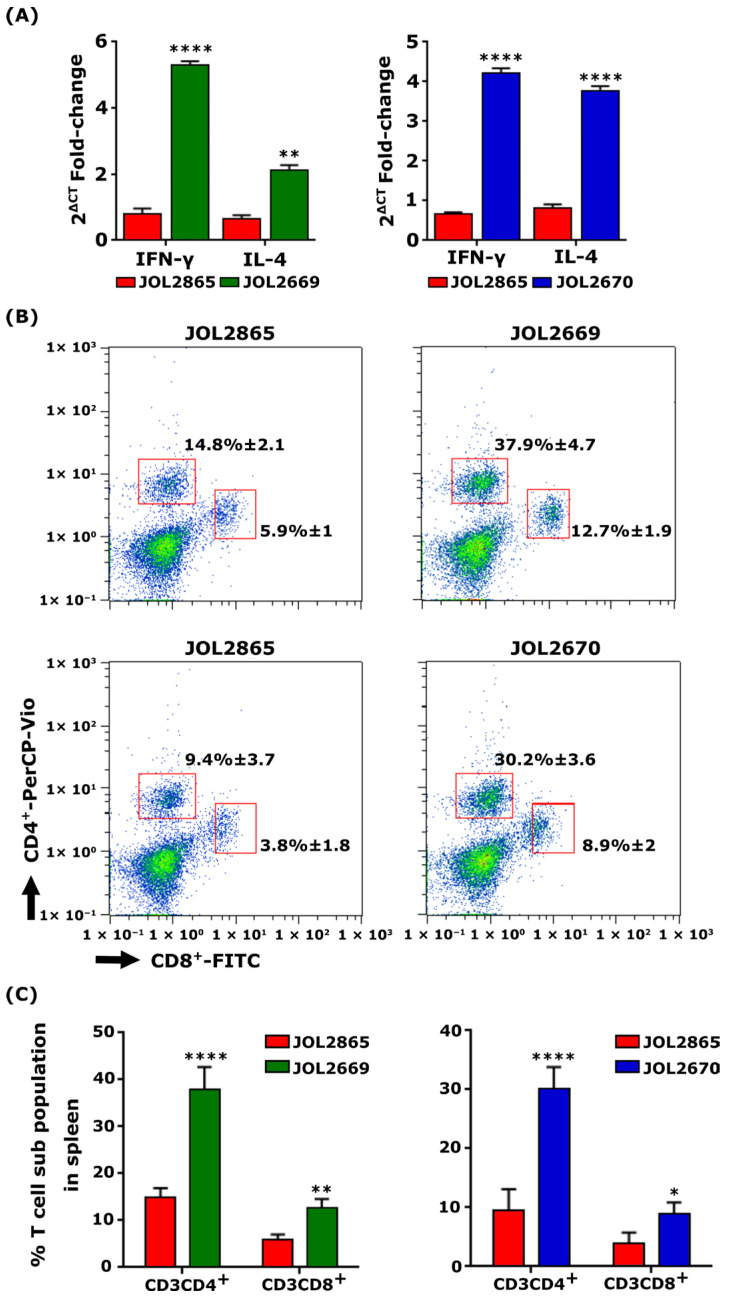
Characterisation of cellular immune responses in immunised mice. (**A**) Fold changes in IFN-γ and IL-4 mRNA levels were determined by RT-PCR at the second week post-booster immunisation. (**B**) Representative flow cytometry dot plots indicating CD4 and CD8 T cell subpopulations in splenocytes collected from immunised mice. (**C**) Quantitative analysis of T cell subpopulations. Statistical significance was analysed using unpaired *t*-tests. * *p* ≤ 0.05, ** *p* ≤ 0.01, **** *p* ≤ 0.0001.

Immunomodulatory cytokines critical for effector T-helper (Th) cell differentiation were evaluated by RT-PCR in splenocytes. Upon antigen re-stimulation, mice vaccinated with JOL2669 exhibited upregulated IL-4 (6.6-fold) and IFN-γ (3.5-fold) mRNA levels compared to the control mice. Similarly, mice vaccinated with JOL2670 elicited higher IL-4 (6-fold) and IFN-γ (4.8-fold) mRNA levels compared to the vector control (Figure 4A). These findings indicate concurrent activation of Th2-mediated humoral immunity and Th1- mediated cellular immune responses, underscoring the vaccine candidate’s capacity to elicit adaptive immune responses essential against PEDV.

### 3.6. Relative Proportions of CD4^+^ and CD8^+^ T Cells After Vaccination with Recombinant S. Typhimurium

Changes in splenic T cell subpopulations on the 14th day post-booster immunisation were assessed by measuring the expression of the T cell surface markers using flow cytometry. Mice vaccinated with JOL2669 and JOL2670 exhibited a marked increase in both CD4^+^ and CD8^+^ T cell subpopulations (Figure 4B). Specifically, in mice immunised with JOL2669, the proportions of CD4^+^ and CD8^+^ T cells significantly increased by 2.5-fold and 2.2-fold, respectively (Figure 4C). These results demonstrate that designed PED vaccine constructs effectively stimulate cell-mediated immune responses, characterised by the expansion of key T cell subsets essential for clearance of intracellular pathogens like PEDV.

### 3.7. Protective Immune Responses Against Salmonellosis

**Figure 5:**
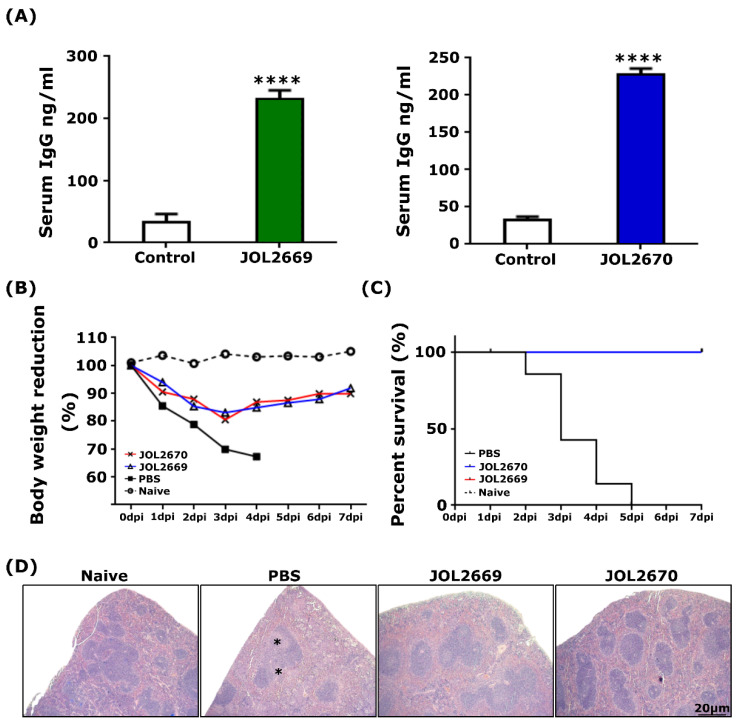
Immunoprotectivity against *Salmonella*. (**A**) Quantification of *Salmonella*-specific serum IgG levels by ELISA at two weeks post-booster immunisation. (**B**) Percent reduction in body weight monitored daily for 7 days following intraperitoneal challenge with virulent *Salmonella* Typhimurium. (**C**) Survival rate of mice was observed from Day 1 to Day 7 post challenge. Immunised groups showed complete protection against lethal *Salmonella* challenge. (**D**) Histopathological analysis of splenic tissue, PBS control mice revealed prominent white pulp atrophy (asterisks). **** *p* ≤ 0.0001.

*Salmonella*-specific IgG antibody levels were significantly elevated at two weeks post-booster vaccination compared to the control group (Figure 5A). The vaccine’s protective efficacy was evaluated by challenging immunised mice with the wild-type *Salmonella* Typhimurium strain. Within five days post-challenge, all unvaccinated mice either succumbed to infection or required euthanasia due to severe disease. In contrast, mice immunised with either the JOL2669 or JOL2670 strains survived the ST challenge with minimal clinical signs (Figure 5B,C). Histopathological examination of splenic tissues from PBS control mice revealed prominent inflammatory white pulp atrophy, whereas tissues from vaccinated mice showed no significant pathological lesions (Figure 5D).

### 3.8. Passive Immunity Acquired by Suckling Mice Against PEDV

**Figure 6:**
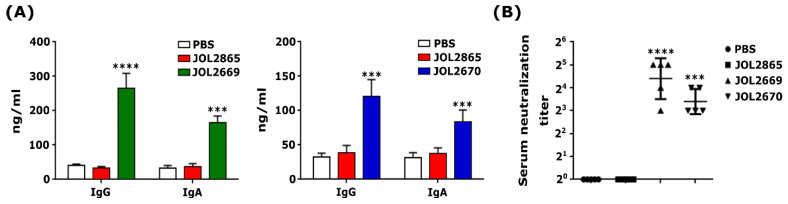
Passive immunity against PEDV is conferred via lactation. (**A**) Antigen-specific serum IgG and intestinal IgA levels were measured in offspring of immunised dams in the 2nd week of postpartum. (**B**) Serum neutralising antibody titres against PEDV assessed by SN assay. *** *p* ≤ 0.001, **** *p* ≤ 0.0001.

Our results demonstrated that suckling mice that received maternal antibodies from dams that were immunised with JOL2669 and JOL2670 showed significantly higher antigen-specific IgG and intestinal IgA titers (Figure 6A). Additionally, serum samples from these offspring displayed markedly higher PEDV-neutralising antibody titers, indicating effective passive transfer of maternal antibodies (Figure 6B). These findings demonstrate that the *Salmonella*-based PED vaccine candidates induced lactogenic transfer of anti-PEDV neutralising antibodies, conferring passive immunity to neonates.

## 4. Discussion

### 4.1. Design and Characteriasation of the SFV-Salmonella-Based PEDV Vaccine Platform

In this present study, we have demonstrated that an attenuated *Salmonella* Typhimuriumstrain delivering an SFV replicon vector expressing PEDV antigens effectively induces an immune response and generates neutralising antibodies against PEDV. Vaccination remains the most effective prevention measure, and both attenuated and inactivated PEDV vaccines have been developed [27,28]. However, genetic and antigenic variations in re-emerging PEDV strains raise concerns regarding the efficacy of commercial vaccines against currently circulating variants [29,30]. The S1 domain of the PEDV spike (S) protein harbours neutralising epitopes and has shown strong cross-protection against PEDV variants [31,32,33]. The PEDV N protein forms a helical nucleocapsid structure and is the most abundantly expressed viral antigen produced in coronavirus-infected cells, thus making it a primary viral target. Additionally, a previous study reported that the N protein induces higher levels of IL-4 and IFN-γ in immunised mice [34]. *Salmonella enterica* serovar Typhimurium has been extensively studied as a live bacterial vaccine (LBV) vector because it can invade intestinal M cells and subsequently infect macrophages, thereby facilitating antigen presentation to the host immune system [35,36]. The attenuation of the live *S*. Typhimurium strain used in this study was derived by deleting three critical virulence-associated genes, *lon*, *cpxR*, and *sifA*, from its genome. Herein, the deletion of the *lon* gene impedes the intracellular proliferation ability of ST, preventing chronic systemic infection, and deleting the *cpxR* from the two-component stress regulatory system *cpxA*/*cpxR* attenuates ST by impairing the bacterium’s ability to respond to membrane stresses, decreasing overall virulence [37,38]. When plasmids are used as delivery vehicles, efficient cytoplasmic release is crucial for successful antigen expression. *Salmonella*’s tendency to persist within the *Salmonella*-containing vacuole (SCV) poses a barrier to plasmid delivery to the host cell cytoplasm and nucleus [39]. However, deletion of the *Salmonella* pathogenicity island 2 (SPI-2) gene sifA disrupts SCV maturation, promoting bacterial escape into the cytoplasm and enhancing delivery of plasmid DNA. In a previous study, we devised a cutting-edge method for generating antigens that leverages the synergies between RdRp from Semliki Forest Virus (SFV) and cytoplasmic mRNA amplification. The well-characterised SFV genome allowed precise identification of RdRp and its sub-genomic promoter region that is necessary for replication and expression. This approach circumvents limitations relating to nuclear plasmid delivery and transcription, enabling high-level antigen expression critical for potent immune stimulation.

### 4.2. Immunogenicity and Protection Efficacy in Mice

In this study, we have selected consensus sequences of S1-NTD (19-223a.a) and N protein from circulating PEDV strains isolated from South Korea in recent years and cloned them into the SFV replicon-based eukaryotic expression vector, pJHL204, and transformed them into an attenuated *Salmonella* strain, JOL2500 (∆*lon*, ∆*cpxR*, ∆*sifA*, and ∆*asd*). Expression of the recombinant PEDV antigen in JOL2500 was validated by Western blot and immunofluorescence analysis. Results of this analysis revealed that following phagocytosis, the pJHL204 vector containing antigens was released into the host cytoplasm and resulted in higher antigen expression (Figure 1). Safety assessment of recombinant *Salmonella* strains is essential in their application in vaccine development and microbial delivery systems, ensuring both host and environmental safety while maintaining their immunogenicity. Safety evaluation of the attenuated *Salmonella* Typhimuriumstrain delivering PEDV antigens demonstrated a markedly improved safety profile (Figure 2). The transient colonisation ensures sufficient antigenic stimulation without compromising host health, which reinforces the feasibility of this platform for further development in clinical vaccine applications. Induction of robust humoral and mucosal immunity is a critical attribute of an ideal vaccine delivery strain for enteric viruses such as PEDV, contributing not only to the resolution of infection but also to the prevention of recurrence [40,41]. As shown in Figure 3, mice vaccinated with recombinant *Salmonella* strains JOL2669 and JOL2670 exhibited significantly elevated serum IgG levels compared to the control groups. The IgG subclasses, IgG1 and IgG2a, serve as markers for Th2- and Th1-type immune responses, respectively [42]. IgG1 and IgG2a antibodies bind to the activatory Fc-γ III and Fc-γ IV, which elicit Th2- and Th1-type immune responses, respectively [43]. In this current study, compared to the vector control group, IgG1 and IgG2a responses were markedly augmented in mice vaccinated with JOL2669 and JOL2670 (Figure 3), indicating balanced mixed Th1/Th2 immune responses. Furthermore, the serum neutralisation assay demonstrated that antibodies elicited in vaccinated mice effectively neutralised PEDV infectivity in Vero cell culture, whereas control groups failed to inhibit the virus replication, indicating that JOL2669 and JOL2670 have high antigenicity against PEDV (Figure 3).

Cell-mediated immune responses (CMI) are crucial factors that prevent infection by secreting cytokines that enhance the cellular and humoral responses and mediate cytotoxic lymphocyte (CTL) activity [44,45]. PEDV evades host innate immune responses by blocking NF-κB activity [46]. Therefore, an effective PEDV vaccine candidate should be able to induce robust cell-mediated immunity. Upon antigenic stimulation, naïve CD4^+^ T lymphocytes differentiate into Th1 and Th2 subpopulations and promote cell-mediated immunity by cytotoxic activity and humoral immune responses, respectively [47,48]. Our findings demonstrated that mice immunised with JOL2669 and JOL2670 significantly elevated the CD3^+^CD4^+^ T cell population (Figure 4), indicating that through the activation of the naïve CD4^+^ T cell subpopulation, JOL2669 and JOL2670 are capable of neutralising PEDV by co-stimulation of B cells and mediating phagocytosis. Additionally, we demonstrated that copy numbers of IL-4 and IFN-γ levels were markedly increased in re-stimulated lymphocytes of JOL2669 and JOL2670 immunised mice (Figure 4). The cytokines IL-4 and IFN-γ are the immunomodulatory markers of Th2 and Th1 responses, respectively [47,49]. These overall results suggest that immunisation with JOL2669 and JOL2670 significantly stimulated the activation and differentiation of CD4^+^ T cells into Th1 and Th2 subpopulations, which enhance cellular and humoral immune responses.

The findings of the present study demonstrated that immunisation with attenuated *Salmonella* Typhimuriumstrains, JOL2669 and JOL2670, induced robust humoral immunity and conferred complete protection against lethal wild-type *Salmonella* challenge. The significant elevation of *Salmonella*-specific IgG antibodies post-booster vaccination indicates that both vaccine strains effectively stimulated the adaptive immune system, resulting in a strong humoral immune response. Serum IgG antibodies play a critical role in opsonization, complement activation, and neutralisation of extracellular pathogens, and their induction is widely regarded as a key correlate of vaccine-mediated protection against *Salmonella* infections [50]. The complete mortality observed in the PBS control group is due to uncontrolled bacterial replication within the reticuloendothelial system, culminating in septicaemia and multi-organ failure. In marked contrast, the survival of vaccinated mice with minimal clinical signs indicated that prior immunisation primed humoral immune responses sufficient to control bacterial dissemination and limit systemic pathology (Figure 5).

### 4.3. Implications for Maternal and Lactogenic Immunity

PEDV primarily causes intestinal infections, with transient viremia detected in the serum of young piglets [51]. The most severe disease manifestations occur in neonatal piglets, highlighting the critical need to focus vaccination strategies on inducing mucosal immunity that protects intestinal enterocytes [52]. This necessitates protective levels of mucosal immunity in neonates at birth and throughout the nursing period. Vaccination of neonatal piglets faces two major challenges: first, maternal antibodies in seropositive sows may inhibit the efficacy of live oral vaccines [53]; second, piglets require approximately three weeks post-vaccination to generate their own protective antibodies [54]. Therefore, eliciting robust maternal mucosal immunity that is transferred to neonates through the lactogenic pathway, colostrum and milk are crucial for the immediate protection of neonates against enteric infections. While IM immunisation is classically associated with systemic immune responses, it robustly elicits distal intestinal sIgA and facilitates lactogenic immunity via polymeric immunoglobulin receptor (plgR) and neonatal Fc receptor (FcRn) pathways [55,56], and IgG crosses the placental barrier or concentrates colostrum via FcRn-mediated transport, conferring passive immunity to suckling pups. Our findings demonstrated that the antibodies produced through intramuscular vaccination were able to cross through the placental barrier or were transferred through colostrum, conferring passive immunity to suckling pups (Figure 6).

## 5. Conclusions

These findings demonstrate that the SFV replicon-based vector system effectively mediates RNA-dependent RNA polymerase amplification of antigen-encoding mRNA, resulting in robust cytoplasmic expression of PEDV-S1-NTD (19-223a.a) and PEDV-N antigens, confirmed by Western blot and immunofluorescence analysis. The attenuated *Salmonella* strain exhibits reduced virulence and an improved safety profile while retaining limited organ colonisation, consistent with its suitability as a vaccine delivery platform. Immunisation with the *Salmonella*-based PEDV vaccine constructs elicited strong humoral immune responses, including PEDV-specific IgG and neutralising antibodies, with evidence of mixed Th1/Th2 polarisation. Vaccinated dams generated PEDV-antigen-specific lactogenic antibodies, indicating the potential for passive transfer of immunity to neonates. Moreover, the attenuated *Salmonella* vaccine conferred significant protection against salmonellosis upon experimental challenge, as reflected by reduced bacterial burden and improved clinical outcomes. Collectively, these results support the feasibility of this *Salmonella* vector expressing PEDV antigens as a multifaceted vaccine candidate. Future studies are warranted to evaluate its protective efficacy against PEDV challenge in swine and optimise dosing and immunisation strategies for field applications.

## Figures and Tables

**Table 1:** List of bacterial strains, plasmids, and primers.

Strains/Plasmids/Primers	Description	References
*S*. Typhimurium		
JOL401	*Salmonella* Typhimurium wild type, SPI-1 invAE+hilA+avr+; SPI-2, amino acid permease+; SPI-3, mgtC+; SPI4, ABC transporter; SPI5, pipB+; antigen preparation	Lab stock
JOL2500	*Salmonella* Typhimurium Δlon, ΔcpxR, Δasd, ΔsifA; bacterial delivery vector	Lab stock
JOL2865	JOL2500 delivering empty pJHL204 vector	Lab stock
JOL2669	JOL2500 delivering pJHL204:PEDS1-NTD	This study
JOL2670	JOL2500 delivering pJHL204: N	This study
*E. coli*		
X232	*E. coli* F^−^ λ− φ80 Δ(lacZYA-argF) endA1 recA1 hadR17 deoR thi-1 glnV44 gyrA96 relA1 ΔasdA4 strain used for cloning of genes into asd+plasmid	Lab stock
BL21pLysS	F–, ompT, hsdSB (rB–, mB–), dcm, gal, λ (DE3), pLysS, Cmr	Progma, USA
Plasmids		
pET28a(+)	IPTG-inducible expression vector; Kanamycin resistant	Novagen, USA
pJHL204	asd+, CMV promoter, SV40 promoter, pBR322 ori	Lab stock
Primers		
IFN-γ FW	TCAAGTGGCATAGATGTGGAAGAA	[25]
IFN-γ RV	TGGCTCTGCAGGATTTTCATG	[25]
IL-4 FW	ACAGGAGAAGGGACGCCAT	[25]
IL-4 RV	GAAGCCCTACAGACGAGCTCA	[25]
β-actin FW	AGAGGGAAATCGTGCGTGAC	[25]
β-actin RV	CAATAGTGATGACCTGGCCGT	[25]

**Table 2:** Summary of immunisation in BALB/c mice. IM, intramuscular; CFU, colony-forming unit; dpi, days post-immunisation.

Group (N = 125)	Strain	Route	Dosage (CFU/100 μL)	Observation	Sample Collection
A (n = 30)	PBS	IM	1 × 10^7^	Mortality and body weight measurement	3, 5, 7, 14, and 21 dpi
B (n = 30)	JOL401	IM	1 × 10^7^		
C (n = 30)	JOL2669	IM	1 × 10^7^		
D (n = 30)	JOL2670	IM	1 × 10^7^		
E (n = 5)	Naïve	-	-		

**Table 3:** Summary of immunisation in BALB/c mice. IM, intramuscular; CFU, colony-forming unit.

Group (N = 75, n = 15)	Strain	Route	Dosage (CFU/100 μL)	Booster
A	PBS	IM	1 × 10^7^	2nd week post-primary vaccination (1 × 10^7^ CFU/100 μL, IM)
B	JOL2865	IM	1 × 10^7^	
C	JOL2669	IM	1 × 10^7^	
D	JOL2670	IM	1 × 10^7^	
E	Naïve	-	-	

## Data Availability

The datasets analysed during the current study are available from the corresponding authors upon request.

## Supplementary Materials

The following supporting information can be downloaded at https://www.mdpi.com/article/10.3390/v18030375/s1. Table S1: Bacterial Load in Spleen and Liver.
