# Supplementary material for: SFV Replicon Vector Harbouring Porcine Epidemic Diarrhoea Virus Immunogens Delivered by Attenuated Salmonella Typhimurium Induces PEDV Neutralising Antibodies and Lactogenic Immunogenicity in BALB/c Mice
Source: Viruses. 2026 Mar 17;18(3):375. doi: 10.3390/v18030375 (PMC13030594; doi:10.3390/v18030375)
Supplement: Supplementary file 1 [file viruses-18-00375-s001.zip › viruses-4163886-supplementary.pdf]

Supplementary Table S1: Bacterial Load in Spleen and Liver

Time (dpi)	Strain	Spleen (log_{10} CFU)	Liver (log_{10} CFU)
1 dpi	JOL401	4.25±0.26	5.02±0.63
	JOL2669	2.60±0.27	2.71±0.54
	JOL2670	2.69±0.75	2.94±0.42
3 dpi	JOL401	5.53±0.57	6.51±0.08
	JOL2669	3.88±0.28	3.67±0.55
	JOL2670	4.13±0.31	3.88±0.35
5 dpi	JOL401	5.61±0.29	6.28±0.12
	JOL2669	3.56±0.67	2.67±0.50
	JOL2670	3.11±0.96	2.98±0.87
7 dpi	JOL401	-	-
	JOL2669	2.95±0.36	2.00±0.50
	JOL2670	2.61±0.41	2.15±0.12
14 dpi	JOL401	-	-
	JOL2669	n.d	n.d
	JOL2670	n.d	n.d
21 dpi	JOL401	-	-
	JOL2669	n.d	n.d
	JOL2670	n.d	n.d

Data expressed as log_{10} CFU/organ (Mean ± SD), n.d: not detected
